# Androgens alleviate the depression-like phenotype in female mice by inhibiting AVPR1a in the hippocampal brain region

**DOI:** 10.1186/s10020-025-01272-9

**Published:** 2025-05-29

**Authors:** Shimin Ren, Xian Wang, Xueying Huang, Liyang Chen, Bing Zhang, Yang Li, Xin Huang

**Affiliations:** 1 Reproductive Medical Center, Xinhua Hospital, Shanghai Jiao Tong University School of Medicine, Shanghai, 200082 China; 2 School of Chinese Materia Medica, Nanjing University of Chinese Medicine, Nanjing, Jiangsu 210023 China; 3 State Key Laboratory of Drug Research, Shanghai Institute of Materia Medica, Chinese Academy of Sciences, Shanghai, 201203 China; 4 Shanghai Key Laboratory of Maternal-Fetal Medicine, Shanghai Institute of Maternal-Fetal Medicine and Gynecologic Oncology, Clinical and Translational Research Center, Shanghai First Maternity and Infant Hospital, School of Medicine, Tongji University, Shanghai, 201204 China; 5 Key Laboratory of Environmental Medical Engineering and Education Ministry, School of Public Health, Southeast University, Nanjing, Jiangsu 210009 China

**Keywords:** Androgen receptors, AVPR1a, Female, Depression, DHEA, Hippocampus

## Abstract

**Background:**

The prevalence of depression in women is approximately twice that in men. Differences in androgens levels between men and women, due to gonadal differences, may be associated with the development of depression, although the underlying mechanisms are not well understood.

**Methods:**

We evaluated the depressive phenotypes of female mice following low-dose androgen treatment using a variety of behavioral and in vivo electrophysiological experiments. The mRNA profile of hippocampal tissues from female mice treated with dehydroepiandrosterone (DHEA) was constructed through RNA sequencing (RNA-seq). GO and KEGG pathway analyses were performed on the differentially expressed genes. The expression changes of candidate differential genes were verified in hippocampal tissues by quantitative real-time PCR and western blotting. Moreover, the mechanism of action of the DHEA-regulated differential gene (*Avpr1a*), which is involved in the neuroactive ligand-receptor interaction pathway, was determined in vitro.

**Results:**

Chronic DHEA treatment resulted in a distinct antidepressant phenotype and significantly enhanced neuronal excitability of the ventral hippocampal region of female mice. RNA-seq identified the crucial differentially expressed gene, *Avpr1a*. In vitro experiments showed that DHEA reduced levels of the AVP system. Additionally, ChIP-PCR experiments revealed that *Avpr1a* directly targets androgen receptor (AR). Cell function experiments demonstrated that DHEA can inhibit AVPR1a expression through AR in a dose-dependent manner, and this effect can be reversed by the androgen receptor antagonist (Flutamide).

**Conclusion:**

Androgens (DHEA) exert antidepressant effects by inhibiting the binding of *Avpr1a* to AR. The *Avpr1a* gene may serve as a new target for the treatment of depression in women.

**Supplementary Information:**

The online version contains supplementary material available at 10.1186/s10020-025-01272-9.

## Introduction

Depression is a severe, multifactorial, and heterogeneous mental disorder (Ji et al. 2015). It is clinically characterized by a persistent depressed mood, slow thinking, cognitive impairment, and lack of initiative (Peng et al. 2020). According to the World Health Organization (WHO), major depressive disorder (MDD) will be a significant contributor to the global burden of disability and illness by 2030 (Yu et al. 2022; Zhang et al. 2020). Data from epidemiological studies suggest that the prevalence of MDD in women is nearly twice as high as in men, and this proportion continues to increase in older age groups (Kessler et al. 1993; Weissman et al. 1996). Biological processes such as genetic factors, hormonal fluctuations, and sensitivity to hormone levels have been reported to be closely linked to women’s susceptibility to depression (Noble 2005). The increased incidence of MDD in women is associated with hormonal changes, primarily during puberty, pre-menstruation, post-pregnancy, and perimenopause (Albert 2015; Halbreich et al. 2000). In fact, before puberty, depression is rare and occurs at similar rates in girls and boys. With the onset of puberty, women are at increased risk of depression (Breslau et al. 2017).

Androgens, as a primary male sex hormone, regulate mood-associated behaviors in both men and women through different mechanisms (Zuloaga et al. 2020). Clinical research has demonstrated that androgen deprivation therapy in patients with prostate cancer significantly increases the risk of depression, suggesting that androgen deficiency may be an essential factor in the development of depression (Kim et al. 2018). The incidence of depression increases significantly with the decline in androgens and possibly other sex hormones during the perimenopausal or postmenopausal period (Lei et al. 2021). Androgens are hormones closely related to maintaining emotional and psychological functions in women, just as in men (Sherwin 1994). Previous studies have found that low doses of testosterone significantly improve depression scores in women with treatment-resistant MDD (Miller et al. 2009) and that mood disorders and depression after ovariectomy can be reversed with testosterone (Shifren et al. 2000). Fluctuations in androgen levels have a significant effect on depression in women. Notably, androgen levels in women are significantly lower than in men, a difference that may be closely related to sex differences in depression (Weissman, et al. 1996). One of the androgens, DHEA, has been reported to be lower in depressed women (Girdler et al. 2012; Morgan et al. 2010). A high cortisol/DHEA ratio may predict major depression in adolescents at risk for major depression (Goodyer et al. 2003). Furthermore, certain clinical trials have found that DHEA supplementation may have antidepressant potential (Wolkowitz et al. 1999).

The hippocampus, especially in women, is associated with the ability to regulate emotions (Kong et al. 2014). Increased excitability in this brain region has been reported to be essential for mediating the development of depression (Williams et al. 2022; Zhuang et al. 2024). As a neurosteroid, DHEA has a strong biological effect even at very low concentrations and has been shown to have dramatic effects on the growth of cortical neurites and synapse formation in hippocampal neurons (MacLusky et al. 2004; Mellon 2007). Androgens, including DHEA, play a role in the hippocampus by enhancing synaptic plasticity and various synaptic transmission functions, thereby affecting neuronal function and neuroplasticity (Benice et al. 2009; Li et al. 2015; Mukai et al. 2006). DHEA exerts neurobiological effects in hippocampal brain regions primarily through various genomic and non-genomic mechanisms by binding directly to androgen receptors (AR) (Clark et al. 2023). AR is expressed at high levels in several brain regions critical for mood regulation, including the hippocampus, and levels of AR in the brain are regulated by androgens (McPhaul et al. 2001; Simerly et al. 1990). In addition, it has also been reported that DHEA not only affects the levels of monoamine neurotransmitters (dopamine, norepinephrine, serotonin, etc.) but also play a role in balancing the function of arginine vasopressin (AVP) and oxytocin (OT) systems, thereby regulating mood and stress (Sheng et al. 2021). Stress-induced imbalances in the OT/AVP system have been shown to increase the risk of various mental disorders, including depression, schizophrenia, and autism (Abramova et al. 2020). In the ventral hippocampus, AVP is a crucial factor influencing mood and cognition (Neumann et al. 2012). The regulatory effect of AVP on the hypothalamic-pituitary-adrenal (HPA) axis suggests that AVP receptor (AVPR) modulators could be used to treat various central nervous system disorders, including depression, anxiety, and post-traumatic stress disorder (Ryckmans 2010). Based on the hypothesis that hormones interfere with the regulation of endocrine, immune, and neurotransmitter systems, it can be speculated that the prevalence of atypical depression in women is also related to the role of these neurotransmitter (Angst et al. 2002; Anthony et al. 2016; Silverstein 2002). However, it is unclear whether the mechanism by which DHEA regulates depression involves these related transmitters and receptors.

In this study, we clarified the antidepressant-like effects of DHEA in female mice and explored the role of neurotransmitters and receptors, particularly the AVPR1a receptor, in the hippocampal tissue mediated by DHEA in the mechanism of antidepressant action, providing potential targets for the treatment of depression in women.

## Materials and methods

### Animal housing and drug administration

**Fig. 1:**
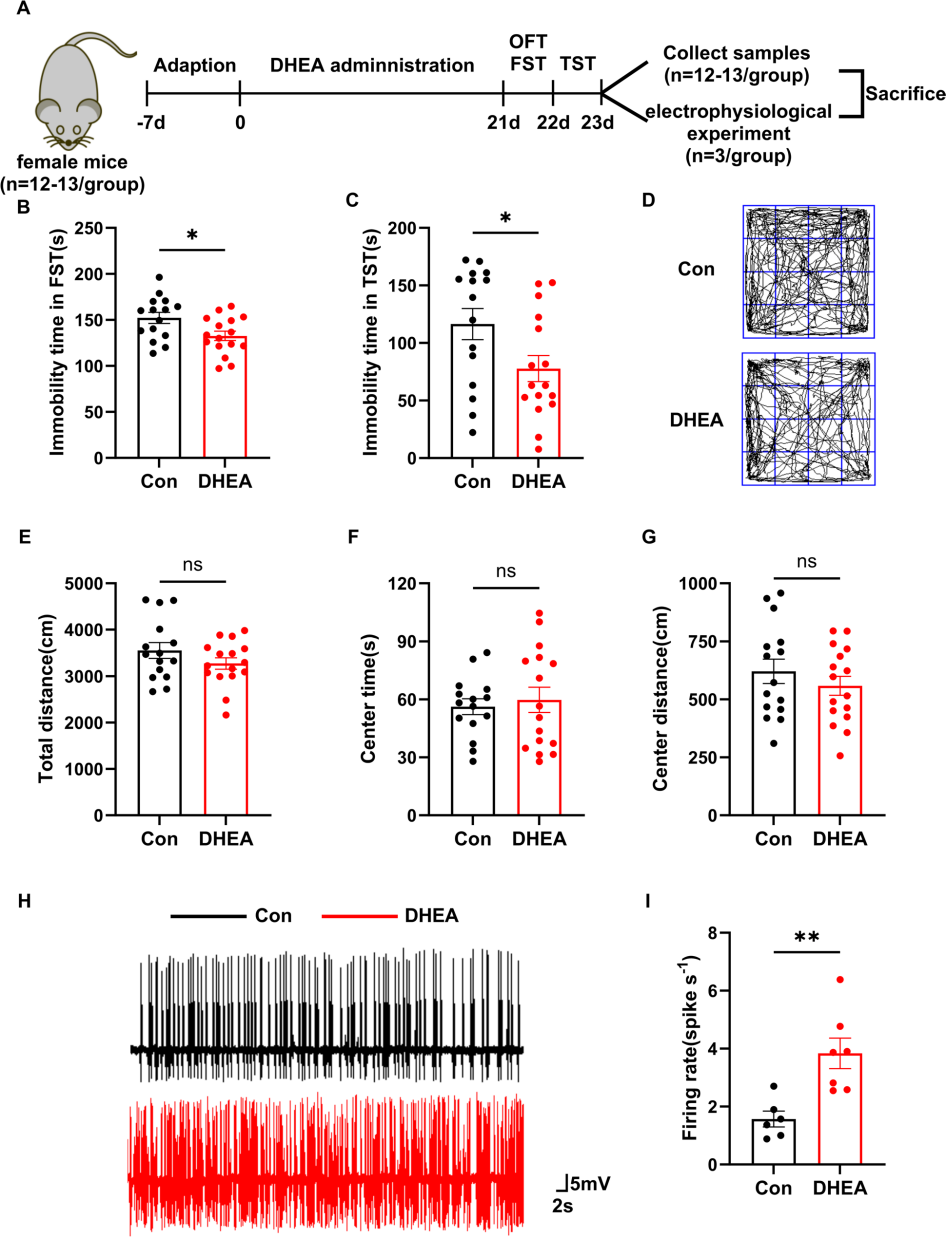
DHEA induces antidepressant-like behaviors in female mice.** A** Experimental design for behavioral tests. **B** In the forced swim test (FST), the immobility time of female mice (15–16 mice per group) after chronic DHEA treatment was significantly lower than that of the control group (15–16 mice per group). **C** In the forced swim test (TST), the immobility time of female mice (15–16 mice per group) after chronic DHEA treatment was significantly lower than that of the control group (15–16 mice per group). **D** Representative locomotion tracks of control and chronic DHEA-treated female mice in the open field test (OFT). **E-G** In OFT, there were no significant differences in total distance (**E**), center time (**F**), and center distance (**G**) between chronic DHEA-treated female mice (15–16 mice per group) and the control group (15–16 mice per group). **H** Representative images of spontaneous firing activities of hippocampal neurons in DHEA-treated and control mice. **I** Firing frequencies of hippocampal neurons in DHEA-treated and control mice. The DHEA group tested 7 neuronal cells from 3 mice, while the control group tested 6 neuronal cells from 3 mice. The data are shown as the mean ± SEM. An unpaired Student’s t-test was used for statistical analysis. * indicates *p* < 0.05, ** indicates *p* < 0.01, ns indicates no significant difference

Because our study focused solely on the impact of androgens on the depressive-like phenotype of female mice, we selected only female mice as experimental subjects. The female C57BL/6 J mice were housed in temperature-controlled rooms with standard 12-hour light/dark cycles, with 5–6 mice per cage, and had access to food and water under specific pathogen-free (SPF) conditions. To minimize age-related variations, we used only 6–8-week-old female mice for this study. The female mice were randomly divided into two groups and received treatment with DHEA (30 mg/kg/day, MCE, #HY-14650) or sesame oil (Beyotime, #ST2627) via abdominal subcutaneous injection. All mice received continuous injections for 21 days (Fig. 1A). In order to exclude the effect of different stages in the estrous cycle on the depressive phenotype of female mice, behavioral measurements were performed during the metestrus or diestrus stages. DHEA-treated mice and control mice (15–16 per group) were all subjected to behavioral tests. After the behavioral studies were completed, 4–5 mice from each group were selected for electrophysiological testing, and the remaining mice were dissected, with hippocampal tissue retained for subsequent experiments, including RNA-seq, qRT-PCR, western blot and ELISA. All animal studies and experimental procedures were approved by the Animal Care Committees of the Shanghai Institute of Materia Medica, Chinese Academy of Sciences (IACUC number: 2024-04-LY-29).

### Cell culture and androgen treatment

HT22 (#CL-0697) is purchased from Procell Life Science & Technology Company (Wuhan, Hubei, China). This immortalized mouse hippocampal cell line has been widely used in the study of various neurodegenerative diseases (Guan et al. 2022; Zhang et al. 2024). The cells were cultured in Dulbecco’s modified Eagle’s medium (DMEM) supplemented with 10% fetal bovine serum and 0.2% penicillin/streptomycin solution in a humidified atmosphere containing 5% CO_2_ at 37 °C (Guan et al. 2022). To determine whether DHEA regulates the expression of AVPR1a protein through AR receptors, the HT22 cells were then exposed to DMSO or DHEA at concentrations of 10 µM or 20 µM for 24–48 h, respectively, along with the androgen receptor antagonist flutamide (10 µM) (Cardounel et al. 1999; Guan et al. 2022; Zhang et al. 2024). In cell function experiments, the cells were pre-treated with flutamide for 1 h before being treated with DMSO or DHEA (Zhang et al. 2024). DHEA and flutamide were dissolved in DMSO, and the control group was treated with an equivalent volume of DMSO.

### Behavior tests

To investigate whether androgen affects the depressant-like phenotypes, the female mice were injected with DHEA (30 mg/kg, sc) for 21 days, followed by behavioral assessments in the following two days. All behavioral tests began at 10:00 am and ended by 6:00 pm. Each group consisted of eight or more female mice, with their littermates serving as controls. Mice were randomly selected for all behavioral tests before grouping. The three standard and classical behavioral experiments conducted in this study were the forced swimming test (FST), the tail suspension test (TST), and the open field test (OFT) (Du et al. 2014; Wang et al. 2015). FST and TST were used to assess depression-like behavior, while OFT was used to evaluate anxiety-related behavior and motor ability in female mice. Before conducting the behavioral tests, mice should undergo acclimatization to the environment to reduce anxiety (Petit-Demouliere et al. 2005). Behavioral testing (OFT and FST) was performed within 24 h, and the TST test was conducted within 48 h after the final dose (Li et al. 2022; Zhang et al. 2022). After the behavioral studies were completed, the hippocampal tissue from each female mouse was collected and divided into left and right portions—half for RNA-seq and half for western blot and ELISA experiments.

### FST

FST was conducted under normal lighting conditions (Castagné et al. 2011). Briefly, mice were individually placed in a transparent cylinder filled with water after three days of acclimatization. The cylinders were sized (35 cm in height ×10 cm in diameter) to prevent the mice from touching the bottom with their limbs. The water temperature was maintained within the range of 22.5–23.5 °C. In order to reduce stress responses during the experiment and help the mice adapt to the experimental environment, mice were allowed to pre-swim for at least 10 min one day before the formal experiment (Petit-Demouliere et al. 2005). During main experiments, mice were allowed to swim for 6 min, and their activities were videotaped throughout. The duration of immobility during the last 4 min was defined as floating or remaining motionless.

### TST

The mice were suspended using medical tape, and their tails were secured to the TST apparatus (Castagné et al. 2011). The experiment lasted 6 min and was videotaped throughout. The duration of immobility during the last 4 min was recorded.

In both the FST and TST experiments, the immobility time refers to the duration during which the mouse stops struggling in water or air. This static state reflects the animal’s “behavioral helplessness”, in which the animal exhibits a sense of helplessness after realizing it cannot escape its current predicament. This is similar to the feelings of helplessness and helplessness seen in human depressive symptoms. Immobility time is an important indicator for assessing depressive-like behavior in animals. In general, the longer the period of inactivity, the more severe the animal’s depressive-like behavior.

### OFT

OFT is a standard method for profiling locomotor activity (Carola et al. 2002). After three days of acclimatization, the mice were placed in the center of a square box (size: 35 × 35 × 45 cm) made of white acrylic plastic. The mice were allowed to explore the box for 10 min and were freely monitored during this period. The movement trail and the total distance traveled during the test were measured using specialized software. The total distance reflects the mouse’s exercise capacity. Center time and center distance are measures of the time and distance that a mouse spends in the center of the open space during the test period. Rodents often exhibit wall-following behavior, i.e., moving along the walls to reduce potential risk. These indicators assess the anxiety level of the mice. The shorter the time the mice spend in the central area, the higher the anxiety level.

### In vivo electrophysiology

To further assess whether DHEA affects the vHPC, we conducted in vivo extracellular electrophysiological recordings on female mice following chronic DHEA treatment to evaluate excitability within the CA1 region of the vHPC. The adult female C57BL/6 mice were administered DHEA and vehicle (sesame oil) for 21 days. In vivo electrophysiological recordings were performed on day 22. Prior to surgery, all mice were anesthetized with pentobarbital sodium (45 mg/kg, i.g.). The mice were then placed in a stereotactic device (Narishige, Tokyo, Japan) for single-unit extracellular experimental recording (Guo et al. 2014). After the mice were stabilized under anesthesia, the skull and dura covering the CA1 region of the ventral hippocampus were removed. The electrodes were pulled from borosilicate glass with an outer diameter (o.d.) of 1.5 mm using a micropipette puller (Sutter, model P-97). Electrodes with a resistance range of 6 to 8 MΩ were filled with 0.9% NaCl and 6% Coomassie brilliant blue (Beyotime, #P0017) solution and then inserted into the CA1 region of the ventral hippocampus (AP: −2.92 mm; ML: ± 3.04 mm; DV: − 2.8 mm) using a manual microdrive. The neuronal discharge was amplified, bandpass filtered with a preamplifier (Axon Clamp,900 A), analyzed with an oscilloscope (Nicolet, Model 2090-I, USA), and stored in a computer equipped with a Clampfit 10.2 (Axon, USA) analysis system for offline analysis. The unit detection range was set to 80 Hz to 10 kHz. To obtain stable neuronal firing in the ventral hippocampal CA1 region, body temperature was maintained at 37 ± 0.5 °C using a thermostatically controlled heating pad (ATC1000; WPI). Three mice per group, with the firing frequency of 2–3 neurons from the hippocampal tissue of each mouse measured separately. And, the recorded neurons were histologically confirmed to be located in the CA1 region of the ventral hippocampus (Fig. S1 ).

### RNA sequencing and differentially expressed gene analysis

RNA-seq was used to screen for genetic changes in hippocampal brain regions after DHEA treatment. Total RNA was extracted from the ventral hippocampal tissue of female mice in the DHEA-treated group (test group, *n* = 4) and the sesame oil injection group (control group, *n* = 4) using TRIzol reagent (Invitrogen, CA, USA) according to the manufacturer’s protocol. RNA purity and quantification were evaluated using the NanoDrop 2000 spectrophotometer (Thermo Scientific, USA). An OD260/OD280 ratio > 1.8 indicates that the absence of protein contamination; an OD260/OD230 ratio > 1.5 indicates the absence of significant contamination from organic substances such as sugars, peptides and phenol. RNA integrity was assessed using the Agilent 2100 Bioanalyzer (Agilent Technologies, Santa Clara, CA, USA). The libraries were then constructed using the VAHTS Universal V6 RNA-seq Library Prep Kit according to the manufacturer’s instructions. Transcriptome sequencing and analysis were performed by OE Biotech Co., Ltd. (Shanghai, China).

The libraries were sequenced on an Illumina NovaSeq 6000 platform, generating 150 bp paired-end reads. Approximately 48.67 million raw reads were generated for each sample. Raw reads in FASTQ format were first processed using FastQC software, and low-quality reads were removed to obtain clean reads. Approximately 46.00 million clean reads were retained for each sample for subsequent analysis. The clean reads were mapped to the mouse genome using HISAT2 software. The FPKM (Fragments Per Kilobase of transcript per Million mapped reads) of each gene was calculated, and the read counts for each gene were obtained using the HTSeq-count.

Differential expression analysis was performed using DESeq2. A p-value < 0.05 and fold-change ≥ 2 or ≤ 0.5 were set as the thresholds for significantly differentially expressed genes (DEGs). Hierarchical clustering analysis of DEGs was performed using R (v 3.2.0) to demonstrate the expression pattern of genes in different groups. Based on the hypergeometric distribution, GO and KEGG pathway enrichment analyses of DEGs were performed to screen significantly enriched terms using R (v 3.2.0). GO analysis was performed to describe the attributes of genes and gene products in any organism (http://www.geneontology.org). This ontology covers three domains: biological processes, cellular components, and molecular functions. The p-value denotes the significance of the GO term enrichment among differentially expressed genes (p-value < 0.05 is considered significant). For KEGG pathway analysis, the web-based Molecular Annotation System 3.0 (MAS 3.0; http://bioinfo.capitalbio.com/mas3/) was used. The significantly altered pathways were selected using the threshold of the p-value and FDR (corrected *p*-value) < 0.05 derived from the hypergeometric test. The DEGs table was listed in Supplementary Table S1 Based on the functional annotations of differentially expressed genes and their fold-change (FC) values, candidate genes potentially associated with depressive phenotypes were selected and further validated using qRT-PCR in additional hippocampal tissue samples.

### Chromatin Immunoprecipitation (ChIP) assay

To verify whether DHEA regulates the expression of the *Avpr1a* gene through androgen receptors (AR), ChIP-PCR was performed to detect the direct binding relationship between the *Avpr1a* promoter and AR protein by using SimpleChIP^®^ Enzymatic Chromatin IP Kit (Magnetic Beads) (#9003, Cell Signaling Technology). The experimental design is shown in Fig. 5A. HT22 cells were cultured in Dulbecco’s modified Eagle’s medium with 10% fetal bovine serum and 0.2% penicillin/streptomycin solution for 5 min. Then, 125 mM glycine was added for 3 min. Briefly, the cells were washed and harvested, then each sample was added with 0.5 µl micrococcal nuclease to obtain DNA fragments of appropriate length and resuspended in sonication buffer and sonicated until transparent, resulting in DNA fragments of 100–500 bp in length. Protein G magnetic beads (Invitro-gen, 10612D) were washed with RIPA 0.3 buffer, and add anti-AR antibody (anti-AR, #ab108341, Abcam) was added. The mixture was rotated at 4 °C for six hours. Then, 1 ml of the samples was mixed with Magnetic Protein G Beads and rotated at 4 °C overnight. The beads were washed with RIPA 0.3 buffer, RIPA 0 buffer, LiCl buffer, and TE buffer, respectively. Immunocomplexes were extracted from the beads with SDS elution buffer containing RNase, followed by Proteinase K. Crosslinks were reversed at 65 °C for at least six hours. Finally, DNA fragments were purified using a DNA purification kit. Five pairs of primers based on the promoter sequence of the *Avpr1a* gene were designed (Supplementary Table S2). The entire experiment was repeated three times, including HT22 cell culture, ChIP and PCR processes.

### Quantitative real-time PCR (qRT-PCR)

The mRNA levels of the *Avpr1a* and AR in the hippocampal tissues and HT22 cells of DHEA-treated and control mice were determined by qRT-PCR. Tissues and cells were homogenized in 1 mL of TRIzol lysate buffer. Total RNA was extracted and reverse-transcribed into cDNA using M-MLV reverse transcriptase (Invitrogen) according to the manufacturer’s instructions. Dye method qRT-PCR quantitative kit (#E21001, GenePharma, Shanghai) were used for qRT-PCR analysis. qRT-PCR was then performed using a real-time fluorescent quantitative PCR instrument (ABI 7500, Thermo, USA). The expression levels of the target genes were normalized to ACTB expression in the same sample (Suzuki et al. 2000) and quantified using the 2^−ΔΔCt^ method (Livak et al. 2001). The components for qRT-PCR; the gene-specific primers and the melting curve were listed in Supplementary Table S3.

### Western blot

The protein levels of the AVPR1a and AR in the hippocampal tissues and HT22 cells of DHEA-treated and control mice were determined by western blot. Hippocampal tissue of female mice (*n* = 3/group) or HT22 cells (*n* = 3/group) were collected. The samples were then homogenized in ice-cold RIPA lysate buffer (96%RIPA, 1% EDTA, 1% PMSF, and 2% protein phosphatase inhibitor). During the protein extraction, 20 µL of protease inhibitor was added to prevent protein degradation. The total protein concentrations were determined using the bicinchoninic acid assay (Pierce, Rockford, IL, USA) for each 50 µL of lysate. The lysate was boiled in 5× denatured loading buffer at 95 °C for 5 min and stored at −80 ℃. Each sample was incubated separately with one of the following primary antibodies: anti-AVPR1a (#BA3463-2, 1:1000, Boster), anti-AR (#ab108341, 1:1000, abcam), or anti-GAPDH (#5174, 1:1000, Cell Signaling Technology). The membrane was washed and incubated with goat anti-rabbit IgG (ab6721, 1:5000, Abcam) as the secondary antibody. A chemiluminescence reaction solution (#170–5061, BIO-RAD) was added, and the samples were exposed to a Chemiluminescent Image Analysis System (#4600SF, Tanon) for visualization. The density of each band was measured by Western blot using Java Image Processing and Analysis (ImageJ) software. Each target protein’s relative expression level was calculated by the ratio of the target protein density to the internal reference protein (GAPDH) density (Wang et al. 2023).

### Enzyme-linked immunosorbent assay (ELISA)

To understand whether the expression changes of AVPR1a affect the levels of the neuropeptide ligands AVP and OT, we measured the levels of AVP and OT in hippocampal tissue using the ELISA method. After chronic DHEA treatment, the female mice (*n* = 7–8/group) were dissected, and fresh hippocampal tissue was homogenized in PBS containing protease and phosphatase inhibitors, then centrifuged at 10,000 × g for 10 min. The supernatant was collected, diluted fivefold, and immediately stored at − 80 °C for later use. AVP (#BY-EM222476, BYabscience, Nanjing, China) and OT (#MM-0276M1, MEIMIAN, Yancheng, China) levels were quantitatively determined using commercial ELISA kits. The colorimetric reaction was measured at 450 nm. Samples were run in triplicate, and the concentrations were calculated based on standard curves. All operations were carried out according to the instructions.

### Estrous cycle assessment

To determine whether continuous low-dose DHEA treatment affects ovarian endocrine function and leads to abnormal ovulation, the estrous cycle of the mice after DHEA treatment was observed. Vaginal smears were taken daily at 10:00 am from the 11 th to the 20 th day after the first day of treatment. Using a fine-tipped glass rod or a moistened cotton swab, gently insert the tip into the mouse’s vaginal opening, and slightly rotate the swab to collect cells from the vaginal canal. Be gentle to avoid injury (Gonzalez 2016). The stage of the estrous cycle was determined by microscopic analysis of the predominant cell type in the vaginal smears following Shorr staining (Cora et al. 2015). The criteria for distinguishing the different stages are as follows: proestrus (P), which consists of round nucleated epithelial cells; estrus (E), which consists of cornified squamous epithelial cells; metestrus (M), which consists of epithelial cells and leukocytes; and diestrus (D), which consists of nucleated epithelial cells with a predominance of leukocytes (Byers et al. 2012).

### Hematoxylin-eosin (HE) staining

To determine whether low-dose DHEA treatment leads to a cyst-like phenotype in the ovaries of mice, HE staining was performed on the ovarian tissues. Ovarian tissues of female mice in the DHEA-treated (*n* = 16) and control (*n* = 15) groups were fixed in 4% paraformaldehyde for 24 h. The tissues were then washed with phosphate-buffered saline (PBS), dehydrated with ethanol, and embedded in paraffin. The ovaries were longitudinally and serially sectioned into 5 μm sections (CM1850, Leica) and stained with H&E. All sections were observed under a light microscope (Nikon Eclipse Ci-L, Japan). The number of luteal bodies and vesicles was counted in the first, fifth, fifteenth, and twentieth sections selected from consecutive ovarian sections (20 consecutive sections from each ovary). The counts were performed by two professional technicians independently, and the average value was taken. This approach minimized subjective differences due to the ovarian section location and between observers. The sections with intact ovarian structure from the DHEA-treated group and the control group were selected as representative images.

### Statistical analysis

All data are expressed as the mean ± SEM. Statistical analyses were performed using GraphPad Prism 10 (GraphPad Software). The unpaired Student’s t-test analysis was used for comparisons between two groups. Multiple comparisons were performed using one-way and two-way ANOVA, and if significant, Tukey’s post hoc test was applied. Each figure legend specifies the statistical test used. Statistical significance was defined as follows: *p < 0.05, **p < 0.01, ***p < 0.001, and **** *p* < 0.0001.

## Results

### DHEA induced an antidepressant phenotype in female mice

In the FST and TST experiments, the immobility time of DHEA-treated female mice was significantly reduced, exhibiting an antidepressant-like phenotype (Fig. 1B-C). The OFT results showed that the total movement distance was not affected by chronic DHEA treatment (Fig. 1D). Additionally, in the OFT, the total distance, the time and distance moved in the central area showed no changes with DHEA treatment (Fig. 1E-G).

### DHEA increased the excitability of the ventral hippocampus (vHPC) in female mice

The in vivo extracellular electrophysiological recordings showed that the total firing rate, which evaluated neuronal activity in the CA1 region of the vHPC, was significantly enhanced after chronic administration of DHEA, compared to the control group (Con) (Fig. 1H-I).

### The critical molecular signatures expressed in the hippocampus of female mice after DHEA treatment

**Fig. 2:**
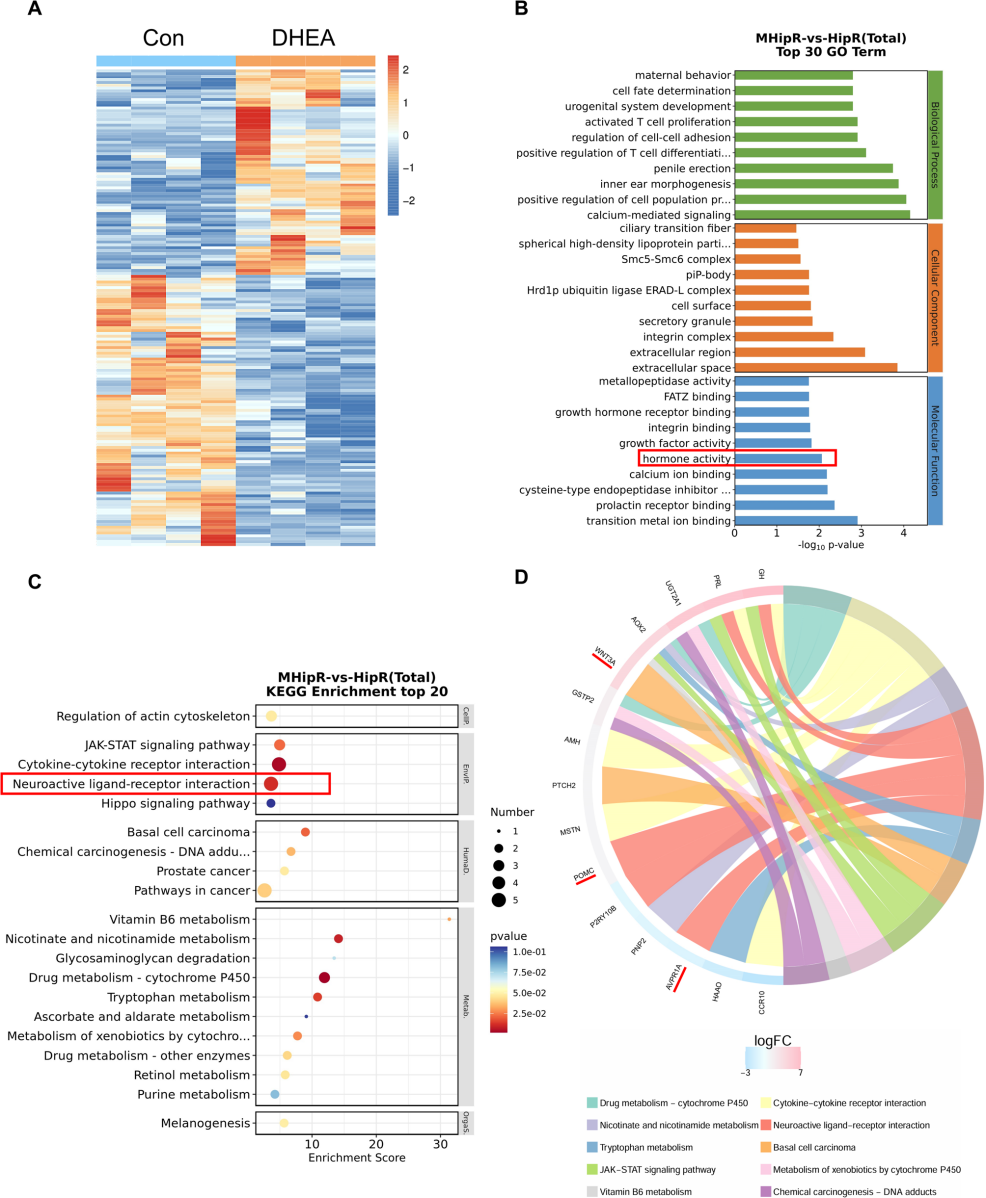
RNA-seq reveals key molecular signatures in hippocampal tissue of DHEA-treated mice. **A** Cluster heatmap of differentially expressed genes between the control and DHEA-treated groups. **B** The total GO term analysis (Top 20) revealed that hormone activity was prioritized. **C** The total KEGG enrichment analysis (Top 20) showed that neuroactive ligand-receptor interaction was significantly enriched. **D** KEGG enrichment analysis using chord diagrams revealed several genes, such as Avpr1a, in the neuroactive ligand-receptor interaction pathway

A total of 167 differentially expressed genes (DEGs) were identified in the hippocampus of female mice treated with DHEA, of which 95 genes were up-regulated and 72 genes were down-regulated. The cluster heatmap of differentially expressed genes between the two groups is shown (Fig. 2A). In the GO analysis, hormone activity was prioritized because it demonstrated the reliability of DHEA treatment (Fig. 2B). Additionally, the KEGG analysis revealed that neuroactive ligand-receptor interaction was significantly enriched (Fig. 2C). A chord diagram was used to display several essential genes involved in neuroactive ligand-receptor interaction, including *Avpr1a*; *Gh*; *Pomc*; *Prl*; *P2ry10b*, and others (Fig. 2D). The specific information about DEGs and enrichment analysis is listed in Supplementary Table S3.

**Fig. 3:**
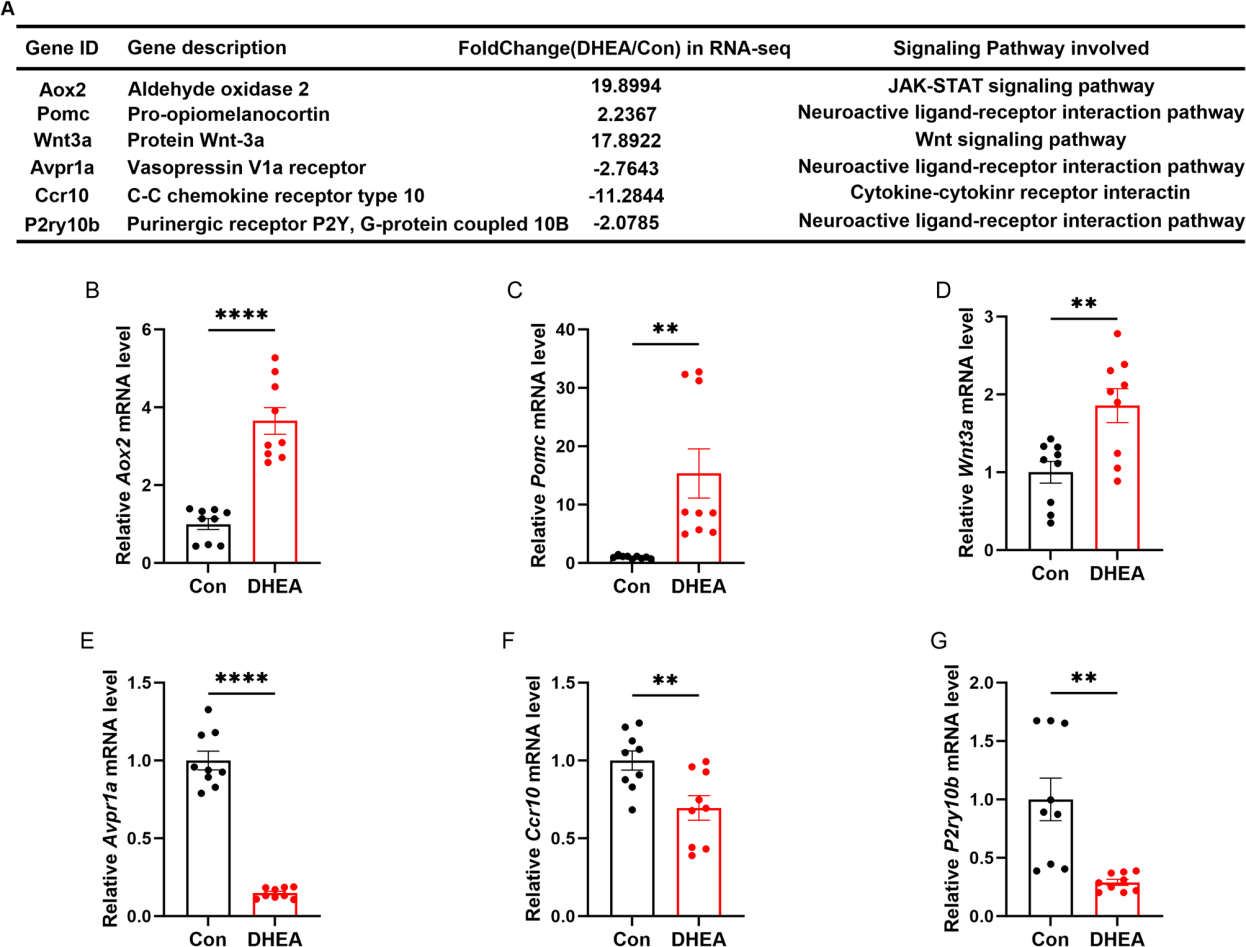
Expression of candidate differential genes was verified by qRT-PCR in female mouse hippocampal tissues.** A** Candidate genes and the signaling pathways they are involved in. **B-G** mRNA expression levels of *Aox2*, *Pomc*, *Wnt3a*, *Avpr1a*, *Ccr10*, and *P2ry10b* as determined by qRT-PCR (*n* = 9). The data are shown as the mean ± SEM. An unpaired Student’s t-test was used for statistical analysis, and the experiment was repeated three times independently with similar results. * indicates *p* < 0.05, ** indicates *p* < 0.01, **** indicates *p* < 0.0001

Based on the relevant functional annotations and their high fold-change (FC) values, six genes (*Avpr1a*,* Pomc*,* P2ry10b*,* Wnt3a*,* Aox2*,* Ccr10*) were selected for validation using qRT-PCR in additional hippocampal samples (Fig. 3A). Of these, three genes (*Avpr1a*,* Pomc*,* P2ry10b*), which are involved in the neuroactive ligand-receptor interaction pathway, were selected. One gene (*Wnt3a*), involved in the Wnt signaling pathway, one gene (*Aox2*), involved in the JAK-STAT signaling pathway, and one gene (*Ccr10*), associated with cytokine-cytokine receptor interactions, were also selected (Fig. 3A). The results showed that all six mRNAs were detected in all hippocampus samples. The mRNA levels of *Aox2*, *Pomc*, and *Wnt3a* were significantly up-regulated by 3.65-fold, 15.33-fold, and 1.85-fold, respectively, in the DHEA-treated mouse hippocampus. The mRNA levels of *Avpr1a*, *Ccr10*, and *P2ry10b* were significantly down-regulated by 6.77-fold, 1.53-fold, and 3.45-fold, respectively. The qRT-PCR results for these six genes were consistent with the RNA sequencing results (Fig. 3B-G), indicating that our mRNA profiling data were reliable.

### DHEA decreased the activity of the AVP system in the hippocampus

**Fig. 4:**
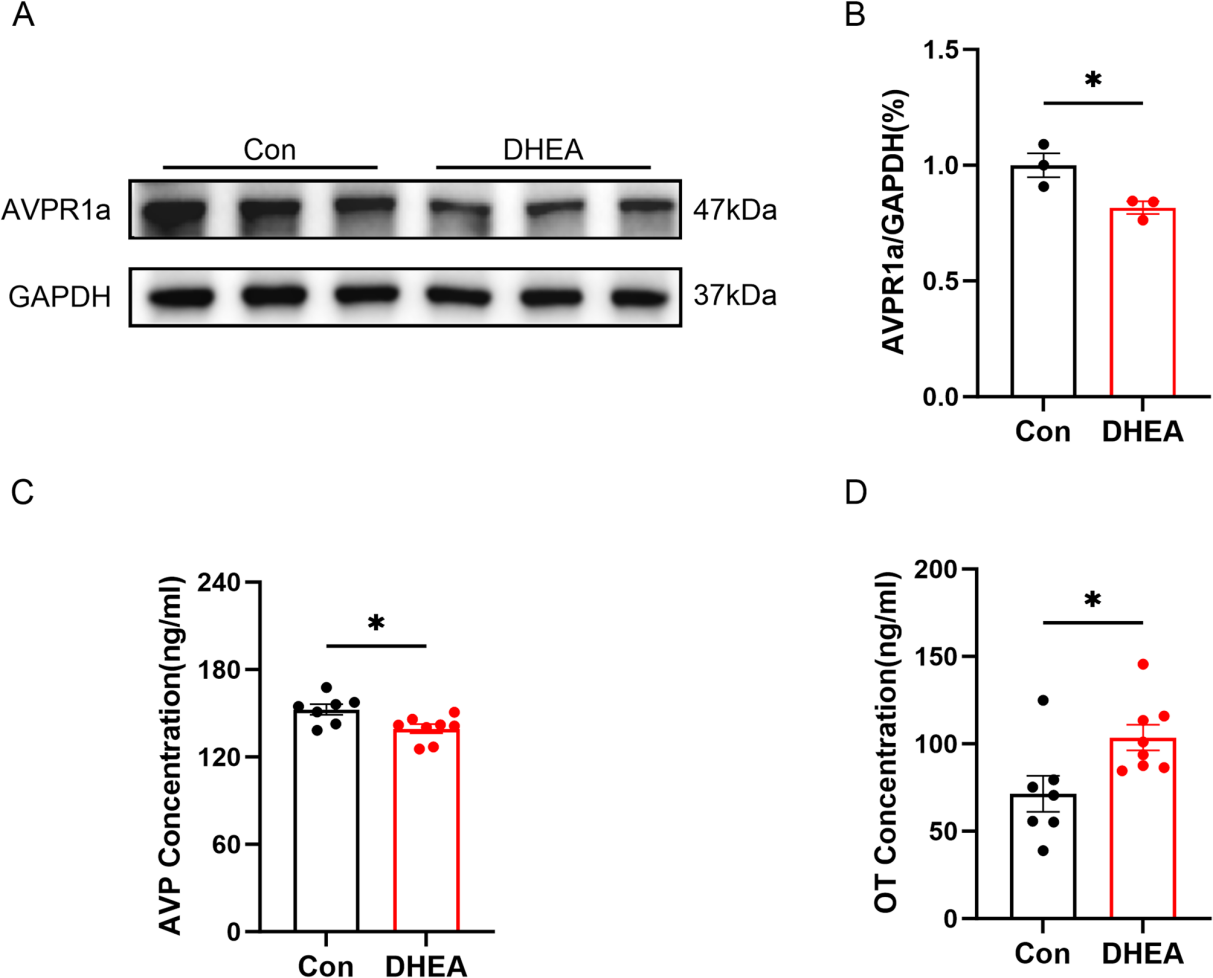
DHEA decreased the activity of the AVP system in the hippocampus. **A** Protein levels of AVPR1a in the hippocampal tissue detected by western blot (DHEA-treated vs. Control, *n* = 3). **B** Relative quantification of AVPR1a protein in the hippocampus of female mice in the DHEA-treated (*n* = 3) and control (*n* = 3) groups. **C-D** The content of AVP and OT in the hippocampus tissues of female mice in the DHEA-treated (*n* = 7–8) and control (*n* = 7–8) groups, as determined by ELISA. The data are shown as the mean ± SEM. An unpaired Student’s t-test was used for statistical analysis, and the experiment was repeated three times independently with similar results, * indicates *p* < 0.05

The AVPR1a protein levels in the hippocampus of DHEA-treated mice were detected by western blot and were significantly lower than those in the control group (Fig. 4A-B). In addition, AVP content in DHEA-treated groups was significantly lower than in the control group, while OT exhibited the opposite trend (Fig. 4C-D).

### ChIP-PCR revealed a direct interaction between the*Avpr1a* and AR

**Fig. 5:**
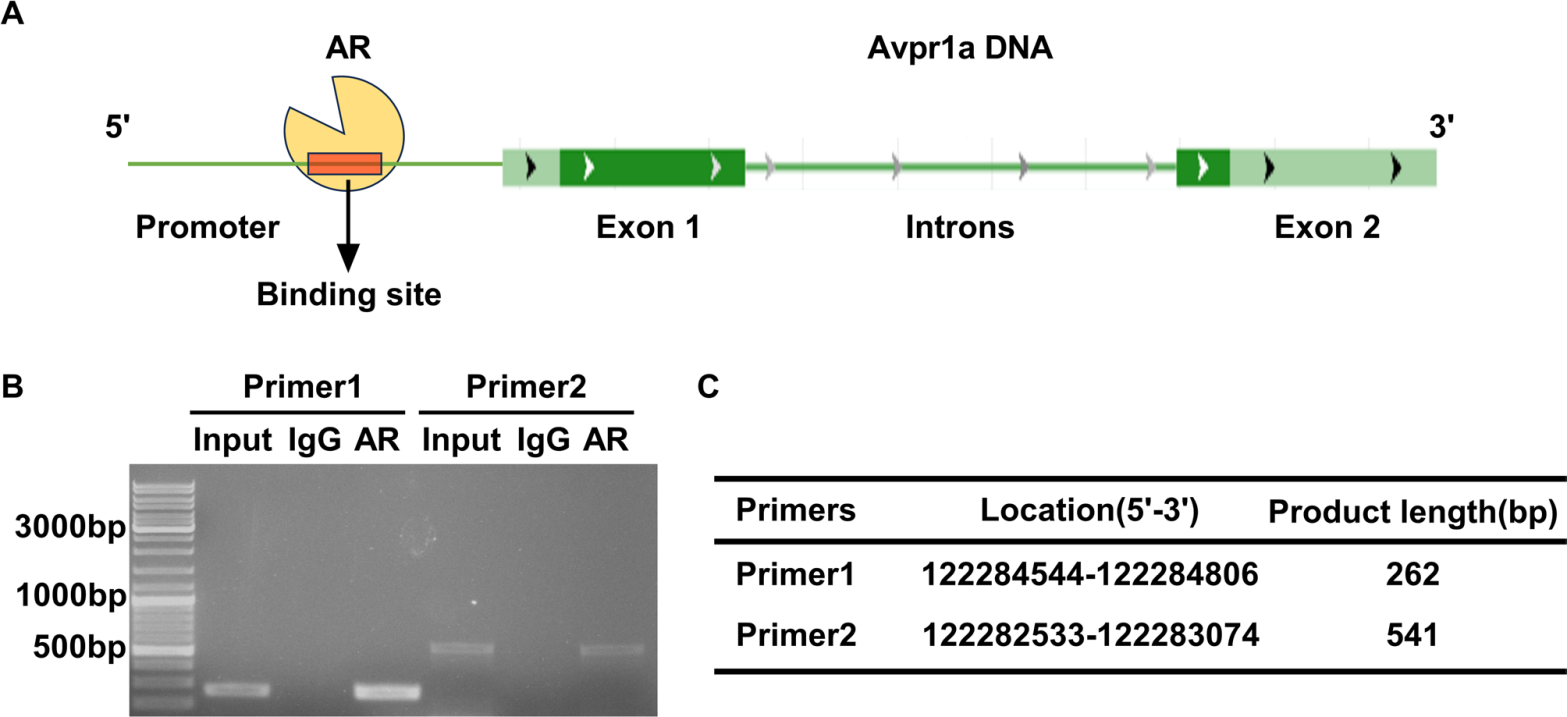
DHEA regulates *Avpr1a* transcription directly through AR.** A** Experimental design for ChIP-PCR. The yellow fan indicates the androgen receptor (AR). The long green line represents the *Avpr1a* DNA. The red rectangle indicates the binding site of the androgen to the *Avpr1a* promoter. **B** Electropherogram of a ChIP-PCR experiment. The bands in the AR group were more intense and more specific than those in the IgG group using the two primers in the PCR process. **C** Sequences of the primers for ChIP-PCR. The experiment was repeated three times independently with similar results

The ChIP-PCR results using five primer pairs targeting the Avpr1a promoter region are shown in Supplementary Fig. S2. Specifically, when two primer pairs (primer 1 and primer 2) targeting the Avpr1a promoter region were used, the AR group exhibited more pronounced and specific bands compared to the IgG group, indicating that the AR protein can directly bind to *Avpr1a* DNA (Fig. 5B-C).

### DHEA inhibited AVPR1a expression in HT22 cells via AR

**Fig. 6:**
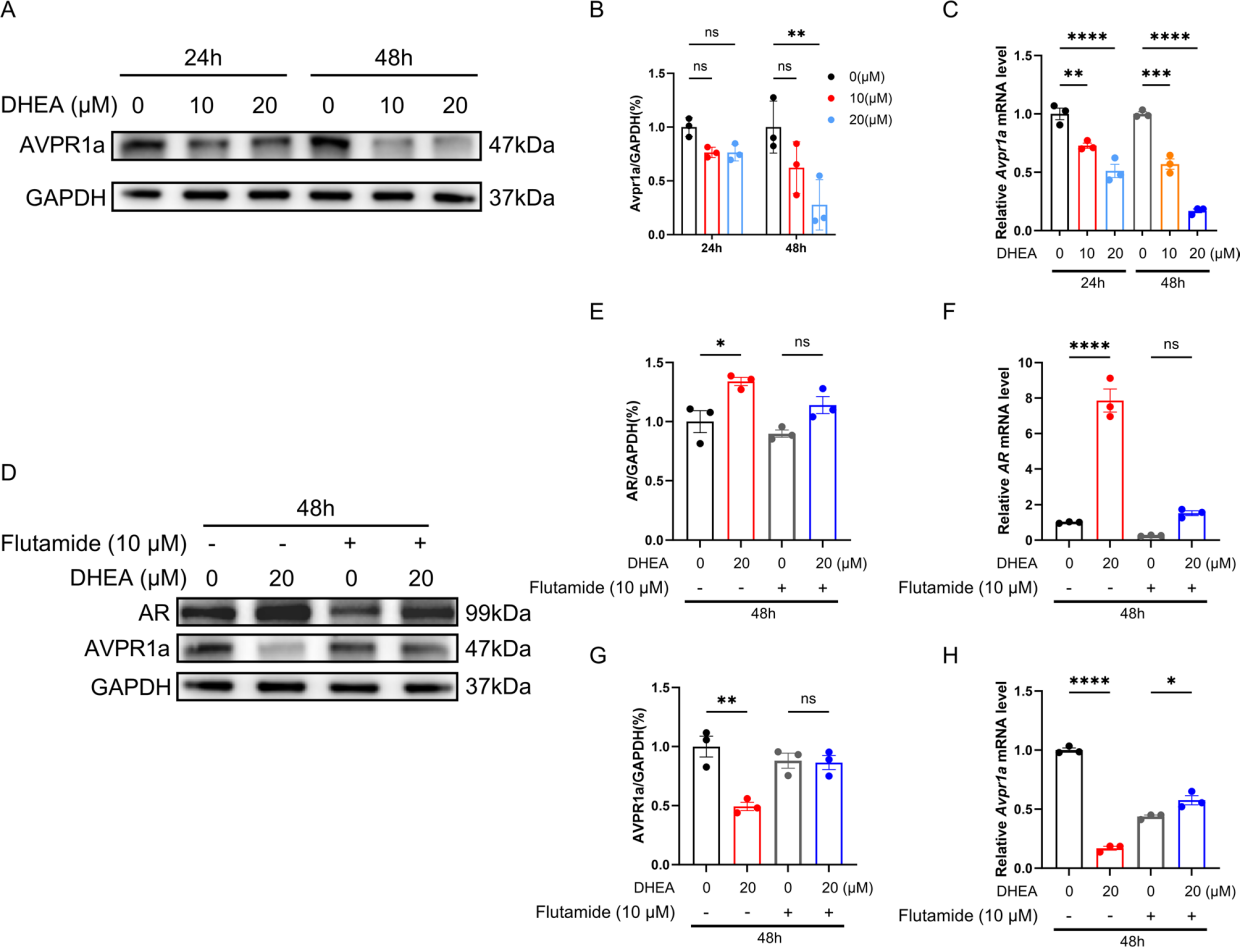
DHEA inhibited AVPR1a expression in HT22 cells via AR. **A** The expression of AVPR1a protein in HT22 cells treated with DHEA at different concentrations (0 µM, 10 µM and 20 µM) for different durations (24 h and 48 h) was detected by western blot. **B**,** C** The relative expression of AVPR1a protein (**B**) and mRNA (**C**) in HT22 cells treated with different concentrations (0 µM, 10 µM, and 20 µM) of DHEA for different periods (24 h and 48 h). **D** The expression of AVPR1a protein in DHEA-treated HT22 cells after antagonist pretreatment was detected by western blot. **E**,** F** The relative expression of AR protein (**E**) and mRNA (**F**) in DHEA-treated HT22 cells after antagonist pretreatment. **G**,** H** The relative expression of AVPR1a protein (**G**) and mRNA (**H**) in DHEA-treated HT22 cells after antagonist pretreatment. The data are shown as the mean ± SEM. Two-way ANOVA followed by Tukey’s post hoc test was used for Fig. 6B-C and one-way ANOVA followed by Tukey’s post hoc test was used for Fig. 6E-F, G-H, and the experiment was repeated three times independently with similar results. * indicates *p* < 0.05, ** indicates *p* < 0.01, *** indicates *p* < 0.001, **** indicates *p* < 0.0001, ns indicates no significant difference

In vitro experiments in HT22 cells showed that AVPR1a expression at both the mRNA and protein levels was significantly downregulated with increasing DHEA concentration and the extended treatment time, demonstrating a clear dose- and time-dependent effect (Fig. 6A-C). Moreover, after treatment with the androgen receptor antagonist (flutamide, 10 µM), the expression of AR was inhibited at both the mRNA and protein levels, while the mRNA and protein levels of AVPR1a increased significantly (Fig. 6D-H). These findings demonstrated that DHEA treatment significantly enhanced AR expression, which subsequently inhibited AVPR1a expression.

## Discussion

Our study first confirmed in female mice that continuous low-dose DHEA treatment can alleviate the depressive phenotype. Before conducting the FST and TST to assess depressive behavior, we performed the OFT on both the DHEA-treated and control mice. The results showed no differences between the two groups in total distance traveled and time spent in the center area, indicating that DHEA treatment did not affect the mice’s motor abilities. This rules out the possibility of false-positive results in the FST and TST due to DHEA treatment influencing the mice’s motor capacity, making our experimental results more reliable and trustworthy. Additionally, the ovaries of DHEA-treated mice showed follicles at different developmental stages and corpus luteum in the ovarian cortex, with a regular estrous cycle, indicating that mice treated with low-dose DHEA could ovulate normally without exhibiting the PCOS phenotype (Fig. S3). This provided the possibility of using low-dose androgen treatment for female depression in the future.

The most important finding of our study is that DHEA may exert its antidepressant effects by downregulating AVPR1a receptors in the hippocampal tissue through AR, thereby increasing neuronal excitability. In this study, we found that the firing frequency of neurons was significantly enhanced in the ventral hippocampus of female mice in the DHEA-treated group, suggesting that DHEA may alter neural activity in hippocampal tissue in response to depressive behaviors. Furthermore, our analysis revealed that several differentially expressed genes regulated by DEHA, including *Avpr1a*, Gh, P2ry10b, Pomc, and PRL, are involved in the neuroactive ligand-receptor interaction signaling pathway(Carrier et al. 2015). These genes have previously been implicated in emotion regulation in multiple studies (Lesse et al. 2017; Qu et al. 2020; Tao et al. 2023). Building on this finding, we subsequently focused on the role of the *Avpr1a* gene, which is involved in the neuroactive ligand-receptor interaction signaling pathway, in regulating depression. Following DHEA treatment, AVPR1a expression was significantly down-regulated, accompanied by a reduction in the ligand AVP and an increase in OT levels in the hippocampus. These changes suggest that DHEA treatment leads to alterations in the OT/AVP system. Chronic stress is known to significantly elevate AVP levels in the hypothalamus of both humans and rodents (Purba et al. 1996). While AVP is primarily synthesized in the paraventricular nucleus and the suprachiasmatic nucleus, its physiological effects are mediated through binding to AVP receptors, which are highly expressed in the hippocampus. Importantly, chronic stress-induced depression-like behaviors have been shown to increase AVPR1a expression in the hippocampus (Lesse et al. 2017). Furthermore, blocking AVPR1a in the ventral hippocampus has been found to alleviate mood disorder behaviors in rats (Engin et al. 2008). These findings underscore the significant role of AVP and its receptors, particularly AVPR1a, in the pathophysiology of mood disorders.

Notably, while the modulation of neurotransmitter receptors by neuroactive steroids is well-established, previous studies have predominantly focused on classical receptors such as NMDA and GABA (Eser et al. 2008; Mifflin et al. 2015). Fewer studies have investigated the role of neurosteroid hormones in regulating the synthesis and release of arginine vasopressin (AVP), with most focusing on the involvement of estrogen receptors in modulating neuronal AVP expression. These studies have identified that AVP-ergic neurons in the paraventricular nucleus (PVN) brain region express estrogen receptor β (ERβ), and that androgen metabolites, such as 3β-diol, can directly influence AVP expression by binding to ERβ. Specifically, 3β-diol enhances the activity of the AVP promoter through its interaction with ERβ, thereby regulating AVP expression (Axelson et al. 1990) (Axelson and Leeuwen 1990). In contrast, our study demonstrates that DHEA down-regulates the activity of the *Avpr1a* promoter by binding to androgen receptor (AR) in vitro in HT22 cells. Additionally, we identified a direct interaction between the *Avpr1a* promoter and AR protein, further elucidating the mechanism by which DHEA modulates *Avpr1a* expression.

It is worth mentioning that some studies have shown that DHEA is often used as a precursor to steroid hormones, which are metabolically converted to testosterone, DHT, and 17ß- estradiol through metabolic pathways, and then act in different physiological processes (Frye 2001; Rosellini et al. 2001). However, our cell-based experiments point to a direct effect of DHEA (since there is no DHEA metabolism in vitro in these experiments) and do not support the role of DHEA’s metabolites. This is clearly different from the conventional view of DHEA’s metabolic activity. We suggest that there may be a more complex mechanism of action for DHEA in vivo, and our current in vitro experimental results cannot fully account for the various regulatory pathways of DHEA in vivo. Therefore, our study raises the possibility that DHEA plays a direct role in brain tissue.

Clearly, another prominent feature of our study is that it focuses on the clinical phenomenon of the high incidence of depression in women. The incidence of depression is generally higher in women, and DHEA is known to have antidepressant properties. However, many related studies in humans and rodents fail to distinguish between sexual dimorphism (Peixoto et al. 2018; Wolf et al. 1999). The preclinical studies on depression conducted to date have almost exclusively used male animals (Lima et al. 2022), making it difficult to fully understand the physiological and pathological mechanisms of depression in women. Of course, our study also has certain limitations. Whether low-dose androgen treatment can be used as a clinical approach for treating depression in women, and whether *Avpr1a* can serve as a target for drug development for female depression, both require further extensive clinical research.

## Conclusion

DHEA could induce an antidepressant phenotype in female mice. After continuous low-dose DHEA treatment, the mRNA expression profile related to neural activity in the hippocampal tissue of female mice underwent significant changes, particularly with a marked downregulation of the neuropeptide AVP receptor, AVPR1a. Further in this study demonstrated that DHEA, as a neurosteroid hormone, can regulate the targeted binding of AR protein to the *Avpr1a* promoter. The results of this study suggest that *Avpr1a* may become a potential target for future antidepressant treatment of PCOS.

## Supplementary Information

Supplementary Material 1: Fig. S1. Histological validation of in vivo electrophysiology. Whole brain slices were prepared after recording to confirm that the electrodes were accurately placed in the CA1 region of the vHPC. The red box indicates the location where the electrode was inserted.

Supplementary Material 2: Fig. S2. Electropherograms of five pairs of primers for Chip-PCR experiments. In the ChIP-PCR experiment, five pairs of primers were designed for different sites in the *Avpr1a* promoter region. Among them, the PCR electrophoresis results of the first and second pairs of primers showed that the bands in the AR group were stronger and more specific than those in the IgG group. There was no difference in band strength between the other three pairs of primers in the AR group and the IgG group.

Supplementary Material 3: Fig. S3. The ovarian morphology and ovulation status of female mice after DHEA treatment were assessed by HE staining. (A-B) Representative images of HE-stained ovaries from the control group (*n*=15) and the DHEA-treated group (*n*=16). CL: corpus luteum. CF: cysts. The arrows point to follicles at different stages of development. (C) The number of cystic follicles in the two groups. (D) The number of corpora lutea in the two groups. (E-F) The changes in the estrous cycle between the two groups.

Supplementary Material 4.

Supplementary Material 5.

Supplementary Material 6.

Supplementary Material 7.

Supplementary Material 8.

## Data Availability

RNA-Seq data have been deposited in the GEO under the accession number GSE276198. Raw files supporting our findings are available from the corresponding authors upon reasonable request.

## Abbreviations

AR: Androgen receptors
AVP: Arginine vasopressin
ChIP: Chromatin immunoprecipitation
DEGs: Differential expression genes
DHEA: Dehydroepiandrosterone
ELISA: Enzyme-linked immunosorbent assay
Erβ: Estrogen receptorβ
FC: Fold-change
FST: Forced swimming test
HE: Hematoxylin-eosin staining
HPA: Hypothalamic-pituitary-adrenal
MDD: Major depressive disorder
OFT: Open field test
OT: Oxytocin
PCOS: Polycystic ovary syndrome
qRT-PCR: Quantitative real-time polymerase chain reaction
RNA-seq: RNA sequencing
SPF: Specific pathogen-free
TST: Tail suspension test
vHPC: Ventral hippocampus
WHO: World Health Organization
