# Supplementary figures and images for: Androgens alleviate the depression-like phenotype in female mice by inhibiting AVPR1a in the hippocampal brain region
Source: Mol Med. 2025 May 29;31:210. doi: 10.1186/s10020-025-01272-9 (PMC12121182; doi:10.1186/s10020-025-01272-9)

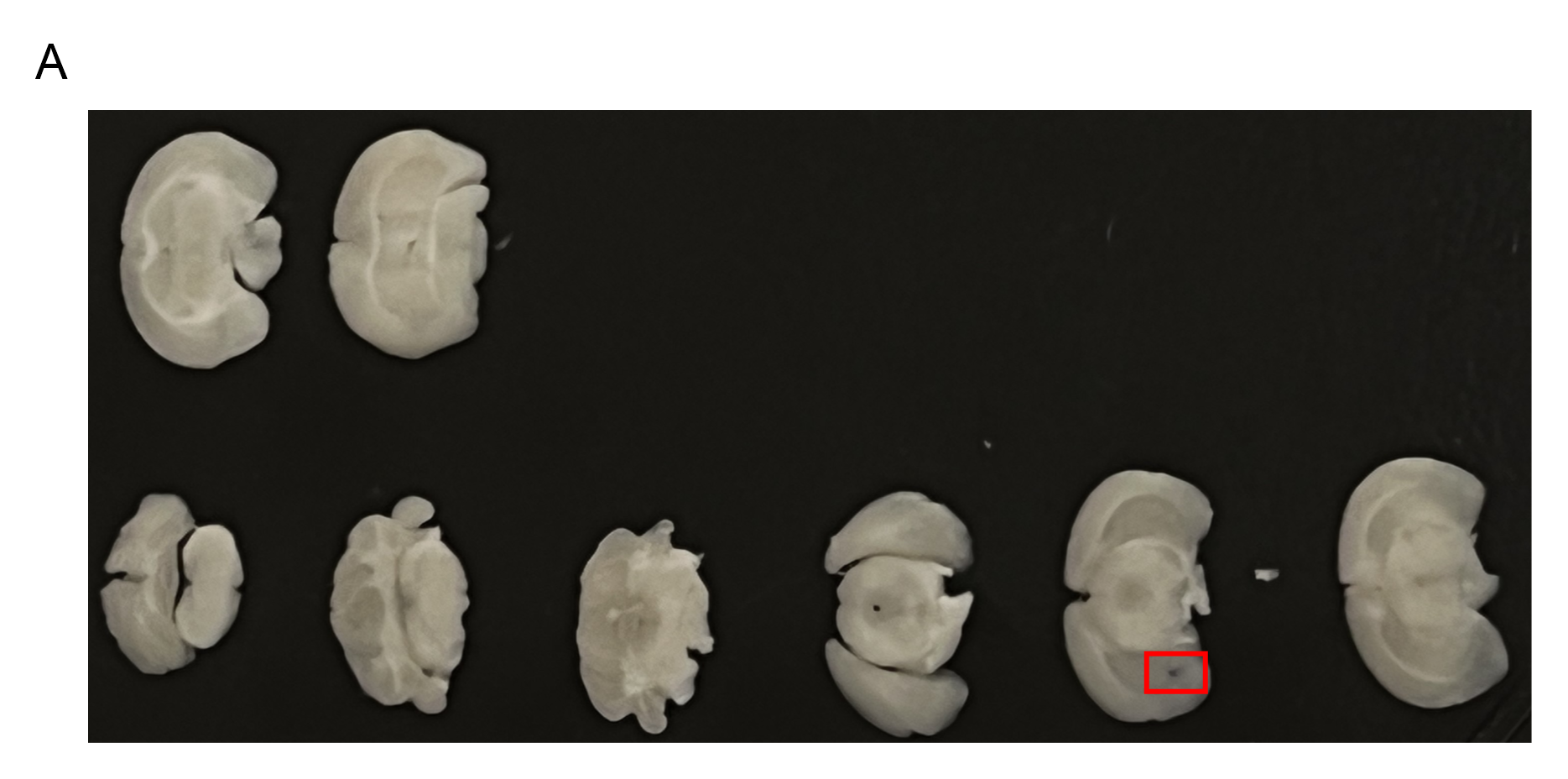

Supplement: Supplementary file 1 — Supplementary Material 1: Fig. S1. Histological validation of in vivo electrophysiology. Whole brain slices were prepared after recording to confirm that the electrodes were accurately placed in the CA1 region of the vHPC. The red box indicates the location where the electrode was inserted. [file 10020_2025_1272_MOESM1_ESM.tif]

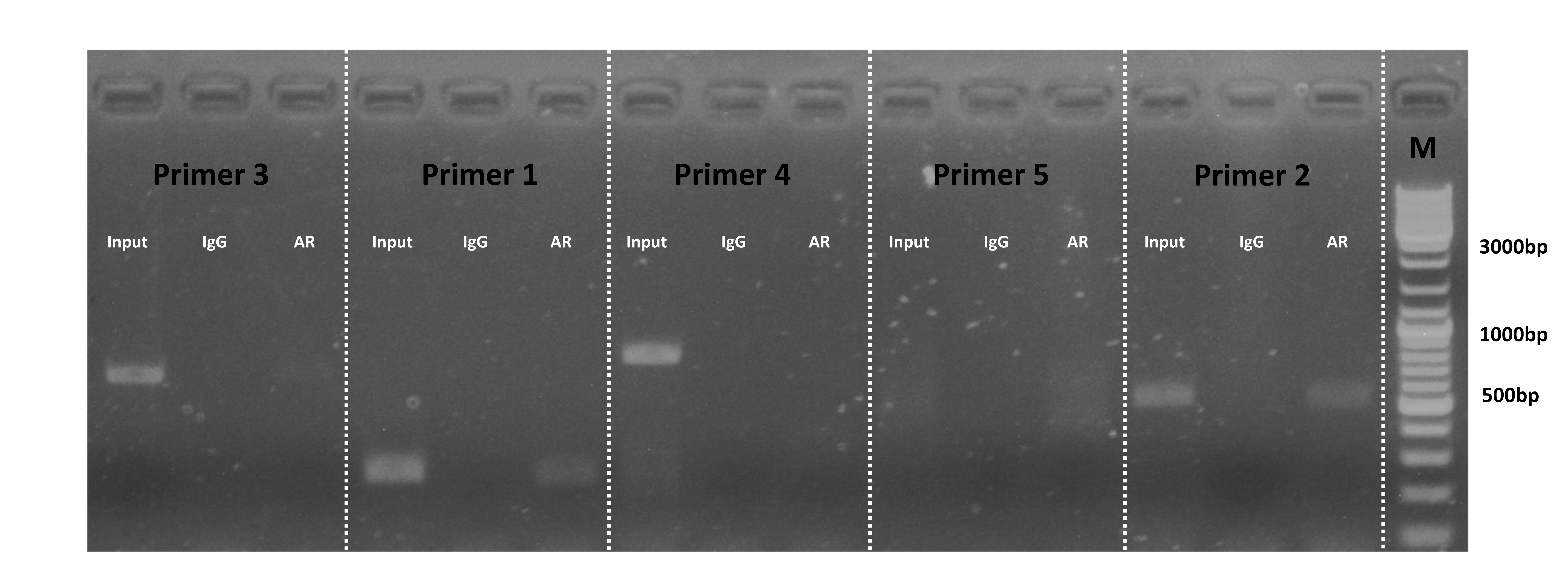

Supplement: Supplementary file 2 — Supplementary Material 2: Fig. S2. Electropherograms of five pairs of primers for Chip-PCR experiments. In the ChIP-PCR experiment, five pairs of primers were designed for different sites in the Avpr1a promoter region. Among them, the PCR electrophoresis results of the first and second pairs of primers showed that the bands in the AR group were stronger and more specific than those in the IgG group. There was no difference in band strength between the other three pairs of primers in the AR group and the IgG group. [file 10020_2025_1272_MOESM2_ESM.tif]

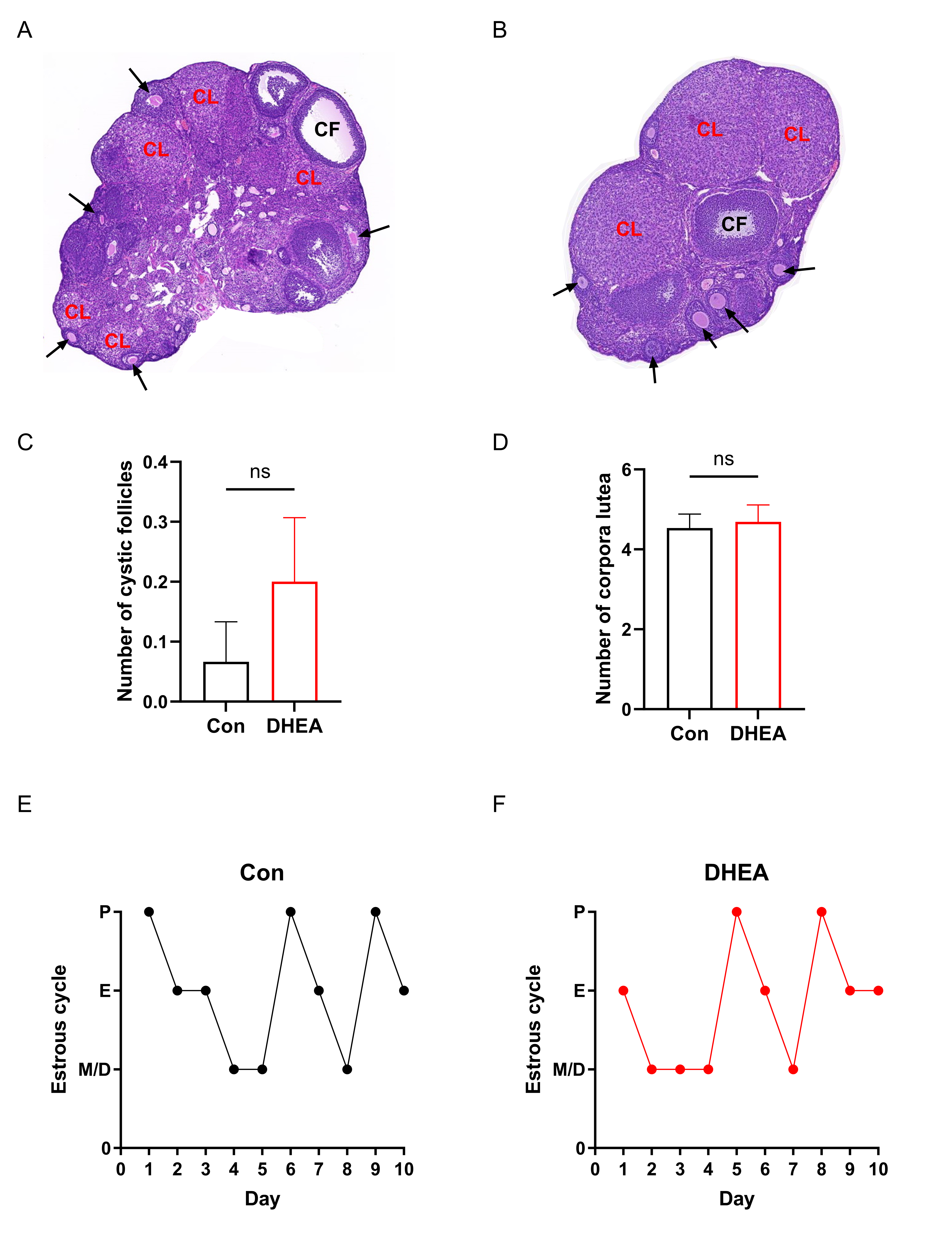

Supplement: Supplementary file 3 — Supplementary Material 3: Fig. S3. The ovarian morphology and ovulation status of female mice after DHEA treatment were assessed by HE staining. (A-B) Representative images of HE-stained ovaries from the control group (n=15) and the DHEA-treated group (n=16). CL: corpus luteum. CF: cysts. The arrows point to follicles at different stages of development. (C) The number of cystic follicles in the two groups. (D) The number of corpora lutea in the two groups. (E-F) The changes in the estrous cycle between the two groups. [file 10020_2025_1272_MOESM3_ESM.tif]

**Fig.4A**

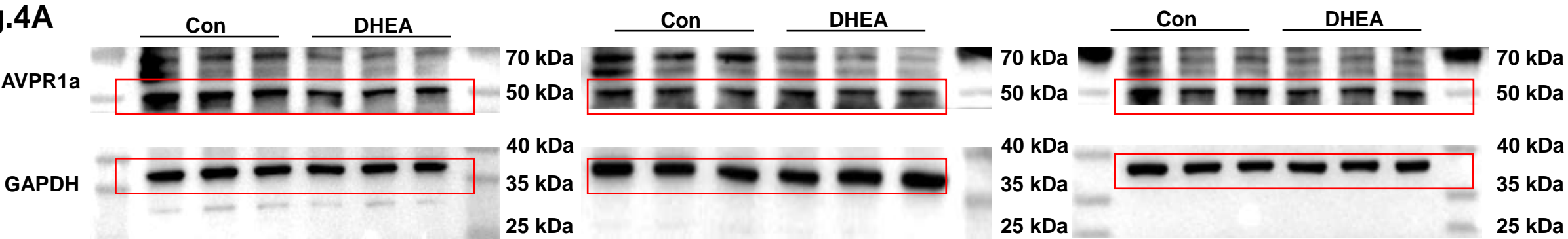

**Fig.6A**

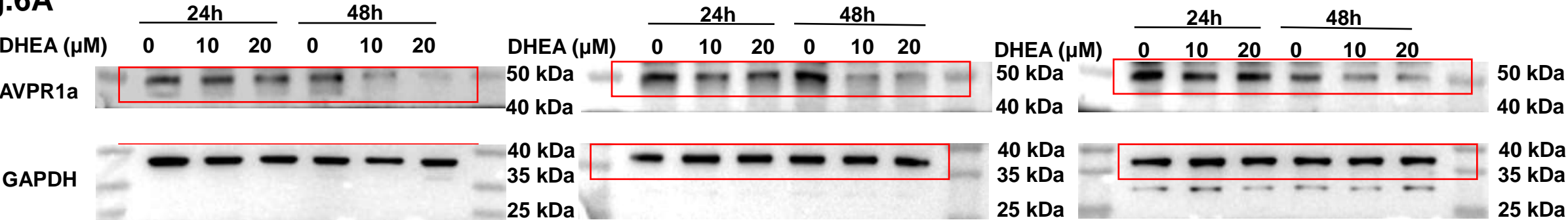

**Fig.6D**

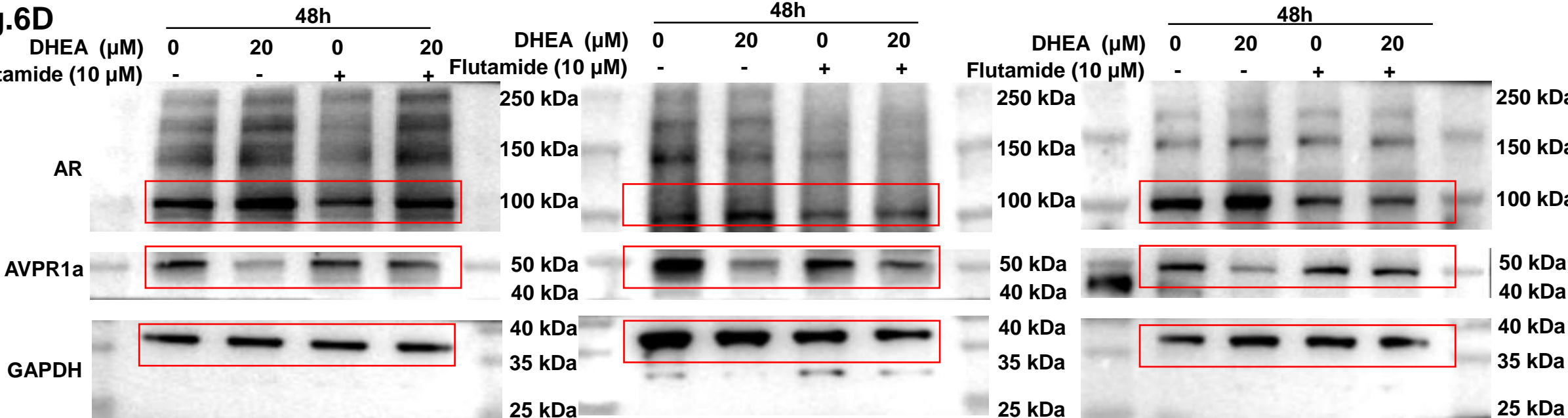

**Fig.5A**

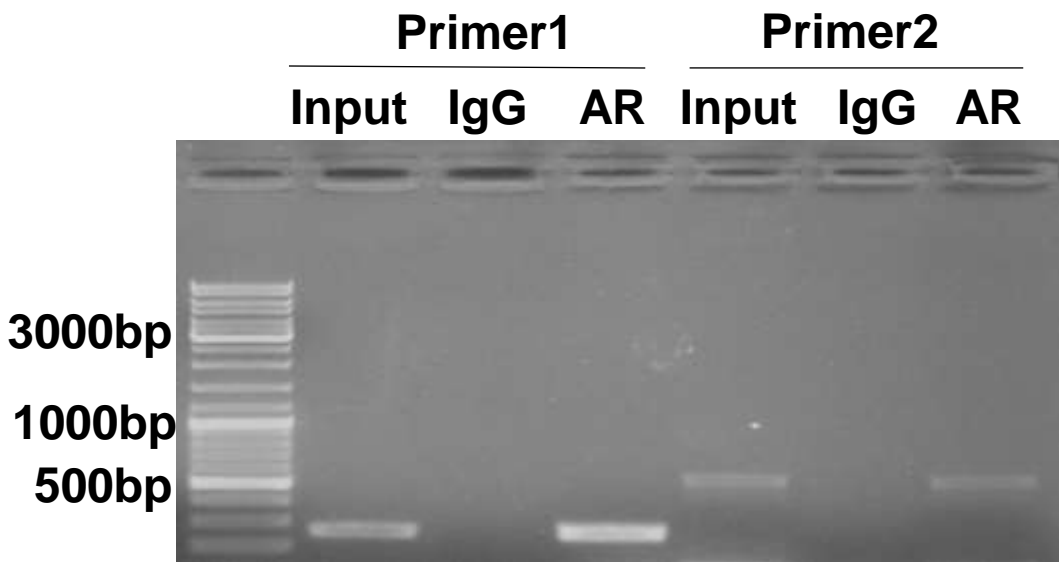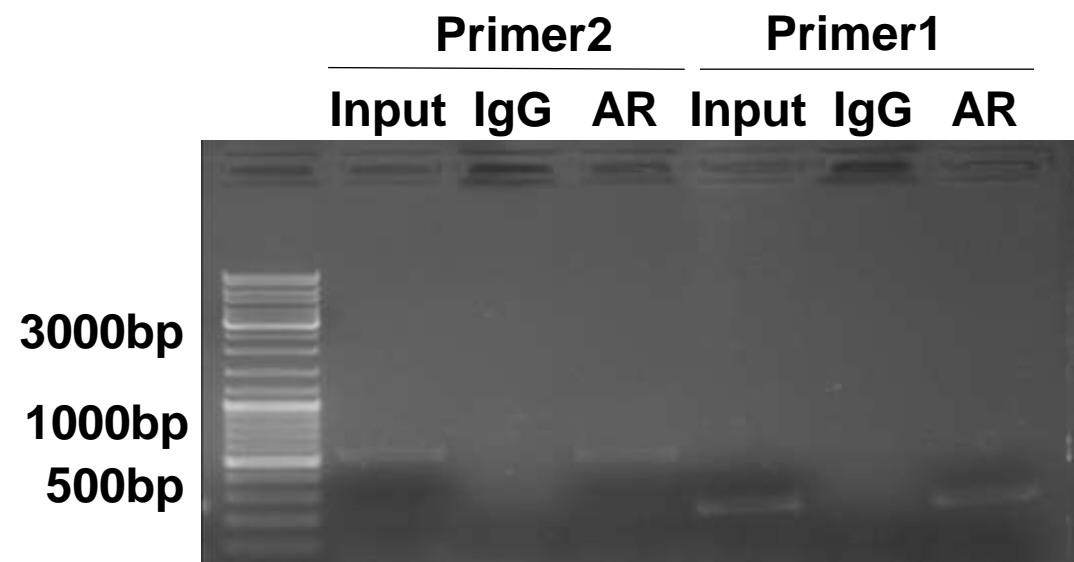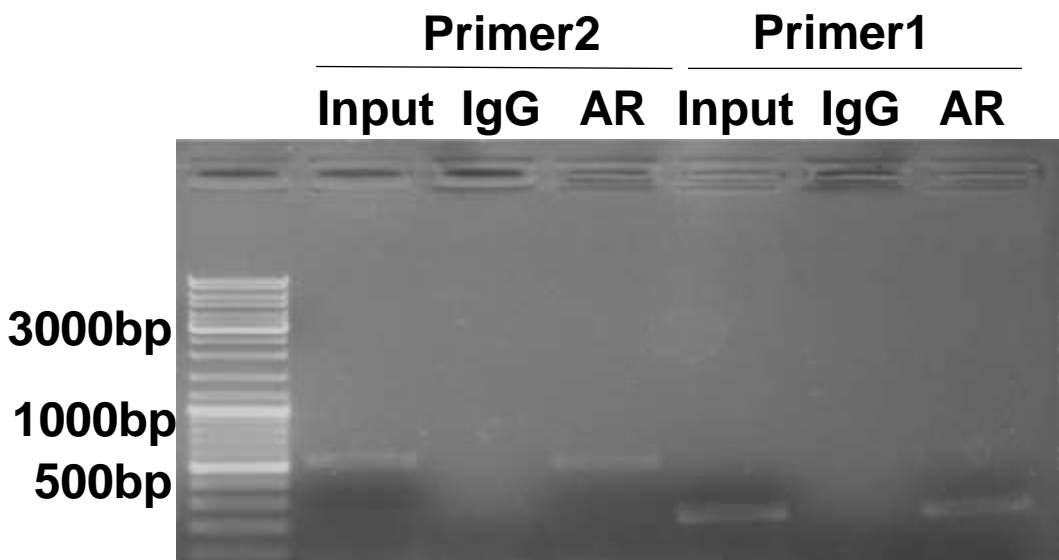

Supplement: Supplementary file 4 — Supplementary Material 4. [file 10020_2025_1272_MOESM4_ESM.pdf]
