# Supplementary material for: Androgens alleviate the depression-like phenotype in female mice by inhibiting AVPR1a in the hippocampal brain region
Source: Mol Med. 2025 May 29;31:210. doi: 10.1186/s10020-025-01272-9 (PMC12121182; doi:10.1186/s10020-025-01272-9)
Supplement: Supplementary file 6 — Supplementary Material 6. [file 10020_2025_1272_MOESM6_ESM.docx]

**Table S2. Primer sequences used for ChIP-PCR**

| **Gene Name** | **Forward Primers (5’-3’)** | **Reverse Primers (5’-3’)** |
| --- | --- | --- |
| Primer1 | 5’-GGGTAGCTGGTAATGTTATTAGTGA-3 | 5’-GTCCACTGGTCAGACAGAGC-3’ |
| Primer2 | 5’-ATGTGTTAATATGGGTAGCTGGTAA-3’ | 5’-TGGAGGATCAAGCAATGCCA-3 |
| Primer3 | 5’-ACACAAAGGTTTGTATGTGTGAA-3’ | 5’-GGCCAGGGACTTGGAATACT-3’ |
| Primer4  Primer5 | 5’-ACACAAAGGTTTGTATGTGTGAAT-3  5’-TGTGTTAATATGGGTAGCTGGTAAT-3 | 5’-TTCTTGGTCAGAGCGTTGGT-3’  5’-AGGAGCTTGACAGCTCTTGG-3’ |
