# Supplementary material for: Androgens alleviate the depression-like phenotype in female mice by inhibiting AVPR1a in the hippocampal brain region
Source: Mol Med. 2025 May 29;31:210. doi: 10.1186/s10020-025-01272-9 (PMC12121182; doi:10.1186/s10020-025-01272-9)
Supplement: Supplementary file 7 — Supplementary Material 7. [file 10020_2025_1272_MOESM7_ESM.docx]

**Table S3. Primer sequences used for qRT-PCR**

| **Gene Name** | **Forward Primers (5’-3’)** | **Reverse Primers (5’-3’)** |
| --- | --- | --- |
| ACTB | 5’ -AAGATCAAGATCATTGCTCCTCC-3’ | 5’ -GACTCATCGTACTCCTGCTTGC-3’ |
| Avpr1a | 5’ -GCCTACATCCTCTGCTGGACA-3’ | 5’ -AAGGAAGCCAGTAACGCCG-3’ |
| AR | 5’- TGCTCCGCCGACATTAAAGA-3’ | 5’-GCTGCTGCCTTCGGAGATTA-3’ |
| Aox2 | 5’-CCAGACGGTTGTCCACAGAA-3’ | 5’-CAAGGGCTGGAACTCGTCTT-3’ |
| Ccr10 | 5’ -ATGTCCAGGCTTTCAGTCGG-3’ | 5’ -GAGGTGGGAGATCGGGTAGT-3’ |
| P2ry10b | 5’ -CAGTGAATCGTGCTTTGCTGA-3’ | 5’ -ACAGGCAATACAAACCCTCCA-3’ |
| Pomc | 5’ -CCTCCTGCTTCAGACCTCCA-3’ | 5’ -GGGGCTGTTCATCTCCGTT-3’ |
| Wnt3a | 5’ -GGCTCCTCTCGGATACCTCT-3’ | 5’ -ACAGCCAAGGACCACCAGAT-3’ |

**The components of qRT-PCR.**

| **Component** | **Final concentration** | | **Amount** |
| --- | --- | --- | --- |
| Real-Time PCR Master Mix | | — | 10μL |
| Upstream primer (20 μM) | | 0.08μM | 0.08μL |
| Downstream primer (20 μM) | | 0.08μM | 0.08μL |
| cDNA template | | — | 2μL |
| Taq DNA polymerase （2.5U/μL）  dd H2O | | 0.05 U/μ  — | 0.4μL  To 20μL |

**The melting curve of qRT-PCR.**

| **Gene Name** | **melting curve** |
| --- | --- |
| ACTB | 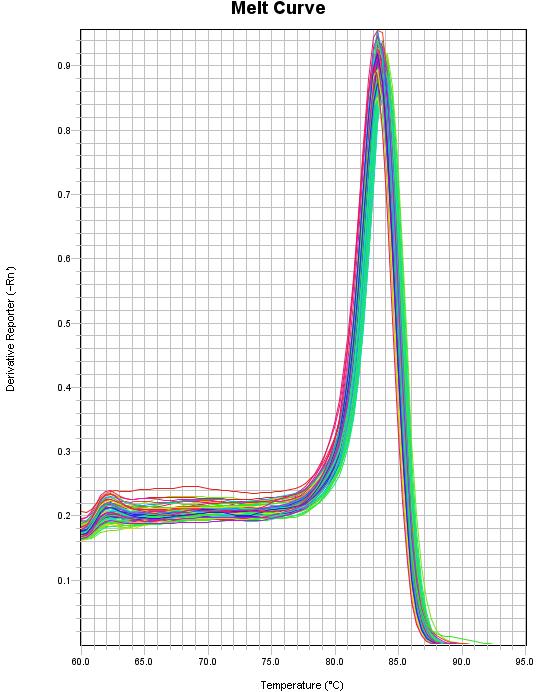 |
| Avpr1a | 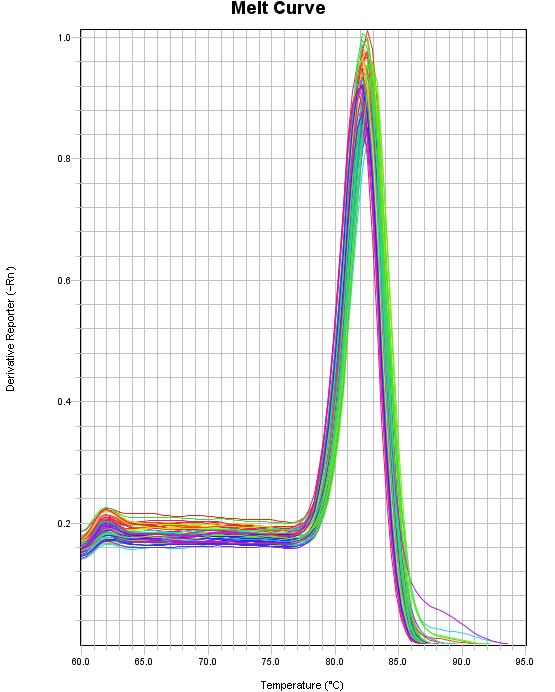 |
| AR | 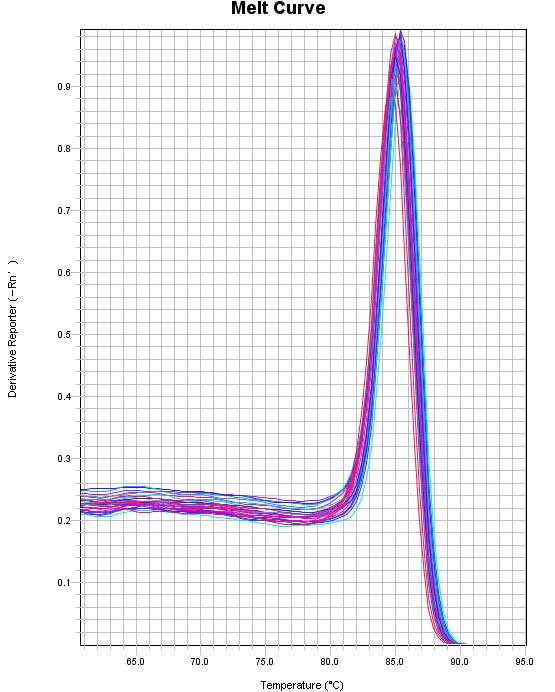 |
| Aox2 | 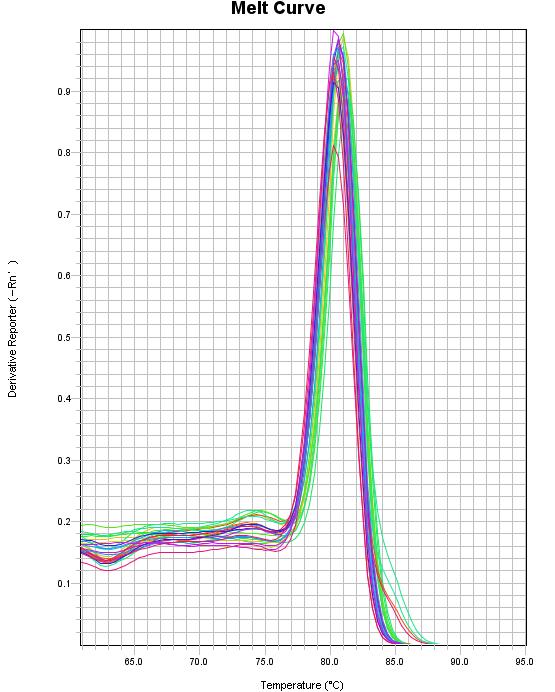 |
| Ccr10 | 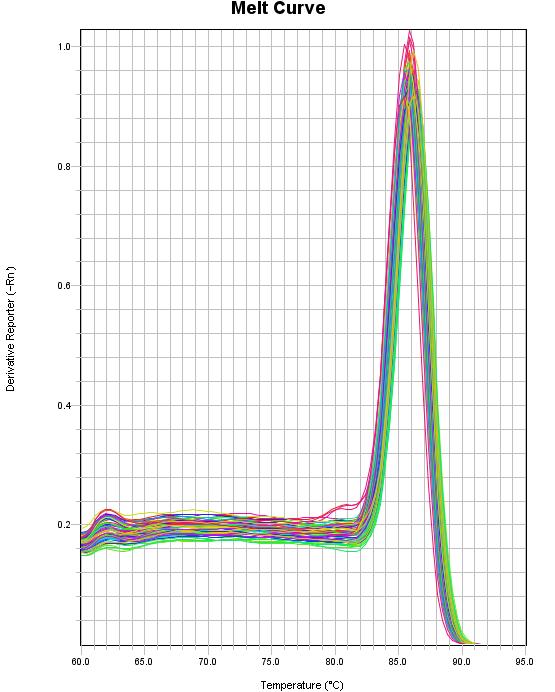 |
| P2ry10b | 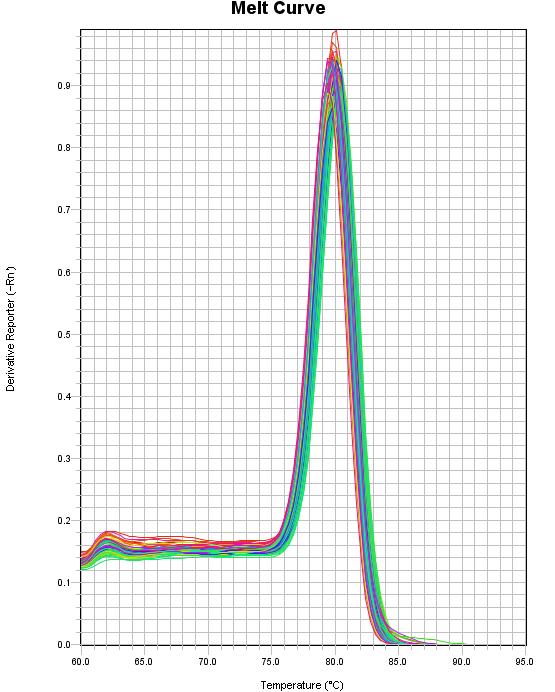 |
| Pomc | 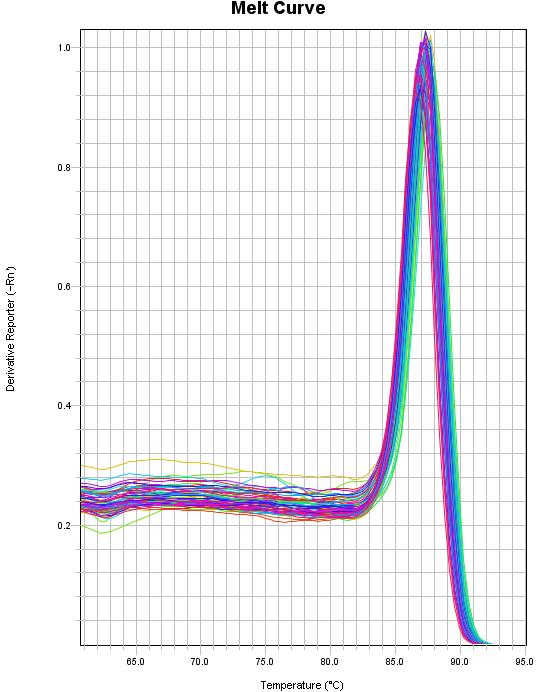 |
| Wnt3a | 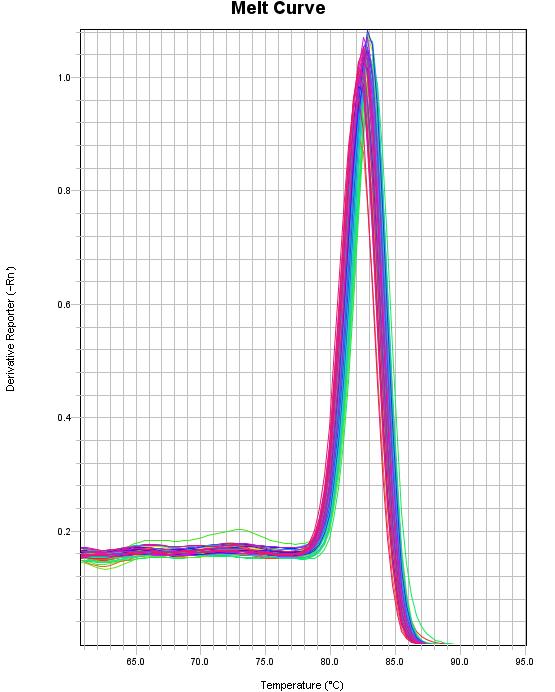 |
